# Synovial tissue response to rituximab: mechanism of action and identification of biomarkers of response

**DOI:** 10.1136/ard.2007.080960

**Published:** 2007-10-26

**Authors:** R M Thurlings, K Vos, C A Wijbrandts, A H Zwinderman, D M Gerlag, P P Tak

**Affiliations:** 1 Division of Clinical Immunology and Rheumatology, Academic Medical Centre/University of Amsterdam, The Netherlands; 2 Jan van Breemen Institute, Amsterdam, The Netherlands; 3 Department of Medical Statistics, Clinical Epidemiology and Biostatistics, Academic Medical Centre/University of Amsterdam, The Netherlands

## Abstract

**Objective:**

To investigate the synovial tissue in patients with rheumatoid arthritis (RA) treated with rituximab and to identify possible predictors of clinical response.

**Methods:**

A total of 24 patients with RA underwent synovial biopsy before, 4 and 16 weeks after initiation of rituximab treatment (without peri-infusional corticosteroids to prevent bias). Immunohistochemical analysis was performed and stained sections were analysed by digital image analysis. Linear regression analysis was used to identify predictors of clinical response.

**Results:**

The 28-joint Disease Activity Score (DAS28) was unaltered at 4 weeks, but significantly reduced at 16 and 24 weeks. Serum levels of IgM-rheumatoid factor (RF) decreased significantly at 24 weeks and anti-citrullinated peptide antibody (ACPA) levels at 36 weeks. Peripheral blood B cells were depleted at 4 weeks and started to return at 24 weeks. Synovial B cells were significantly decreased at 4 weeks, but were not completely depleted in all patients; there was a further reduction at 16 weeks in some patients. We found a significant decrease in macrophages at 4 weeks, which was more pronounced at 16 weeks. At that timepoint, T cells were also significantly decreased. The reduction of plasma cells predicted clinical improvement at 24 weeks.

**Conclusions:**

The results support the view that B cells orchestrate local cellular infiltration. The kinetics of the serological as well as the tissue response in clinical responders are consistent with the notion that rituximab exerts its effects in part by an indirect effect on plasma cells associated with autoantibody production, which could help explain the delayed response after rituximab treatment.

**Trial registration number:** ISRCTN05568900.

Rheumatoid arthritis (RA) is a chronic inflammatory disorder affecting synovial tissue in multiple joints. Early treatment with disease-modifying antirheumatic drugs (DMARDs) has become the cornerstone of therapy. Recently, new biological therapies, including rituximab, have become available. Rituximab is a chimaeric monoclonal antibody directed against the CD20 antigen expressed by B cells, which significantly improves disease symptoms in patients with high levels of disease activity despite treatment with methotrexate (MTX) or tumour necrosis factor (TNF) blockers.^1^^–^3 This clinical effect strongly supports the notion that B cells play a critical role in the pathogenesis of RA, although the exact mechanism of rituximab treatment in RA remains to be elucidated.

We have previously shown that rituximab treatment causes a rapid and specific decrease in numbers of B cells at the primary site of inflammation, the rheumatoid synovium,^4^ which was recently confirmed in another study.^5^ The early synovial tissue response varies between patients, which is in contrast with the marked B cell depletion observed in the peripheral blood of nearly all patients with RA. Interestingly, in the earlier, smaller studies there was no significant decrease in numbers of inflammatory cells other than synovial B cells 4–8 weeks after initiation of treatment.^4^ ^5^

Currently, no data are available on the synovial tissue response to rituximab treatment after more prolonged follow-up and its predictive value related to clinical improvement. The current study was performed to investigate the kinetics of this response in detail and to identify possible predictors of clinical response in patients with RA.

## PATIENTS AND METHODS

### Patients and treatment protocol

A total of 24 patients were included in this study analysing synovial biopsies at three timepoints: before treatment, at 4 weeks and 16 weeks after initiation of rituximab treatment; 17 of these patients participated in a previously reported study on the synovial tissue response to rituximab at 4 weeks only.^4^ The patients had active RA;^6^ active disease was defined as having ⩾4 tender joints and ⩾4 swollen joints of 28 joints assessed, and at least one of the following: erythrocyte sedimentation rate (ESR) ⩾28 mm/h, serum C-reactive protein (CRP) levels ⩾15 mg/litre, or morning stiffness ⩾45 min. In addition, patients needed to be positive for IgM-RF and/or anti-citrullinated peptide antibodies (ACPA) and have active arthritis (defined by the presence of pain and swelling) of a wrist, knee or ankle joint, amenable for arthroscopy.

All study patients were on stable doses of MTX (5–30 mg/week) for at least 28 days prior to enrolment. Stable prednisone therapy (⩽10 mg/day) and non-steroidal anti-inflammatory drug (NSAID) treatments were allowed. All other DMARDs and biological agents were withdrawn at least 4 weeks prior to study inclusion, with a washout period for leflunomide, infliximab, adalimumab and etanercept of >8 weeks prior to randomisation. The study protocol was approved by the Medical Ethics Committee of the Academic Medical Center/University of Amsterdam, and all patients gave written informed consent before participation in the study.

Treatment consisted of two infusions of 1000 mg of rituximab (Roche, Woerden, The Netherlands) on days 1 and 15 after premedication with 2 mg clemastine fumarate intravenously and 1000 mg acetaminophen orally. Peri-infusional treatment with corticosteroids was not allowed, as this could have influenced the features of synovial inflammation.

The 28-joint Disease Activity Score (DAS28)^7^ was measured every month after treatment. Serum levels of IgM-rheumatoid factor (RF) and ACPA (anti-CCP2 ELISA, Immunoscan RA, Mark 2, Euro Diagnostica, Arnhem, the Netherlands) were determined at baseline and weeks 4, 16, 24 and 36 after treatment. Synovial biopsies were obtained from the same clinically involved joint at three timepoints: before as well as 4 and 16 weeks after the first infusion of rituximab.

### Blood lymphocyte populations

Flow cytometry on fresh peripheral blood samples was performed at baseline and at 4, 16 and 24 weeks after the first rituximab infusion. B cells and T cell subsets were detected by real-time fluorescence-activated cell sorting (FACS) using a FACSCalibur Flow Cytometer (Becton Dickinson, San Jose, California, USA) with antibodies against CD19, CD3, CD4 and CD8 (all from Becton Dickinson). CD19 was chosen because of its similar expression to CD20 on B cell subsets, without interference with the circulating rituximab antibody. The lower limit of detection of CD19+ B cells was set at 0.01×10^9^ cells/litre.

### Synovial biopsy

Serial synovial biopsies were collected by needle arthroscopy from the same actively inflamed joint under local anaesthesia as previously described,^8^ before as well as 4 and 16 weeks after the first infusion of rituximab. To minimise sampling error at least six biopsy samples were obtained from different sites in the joint during each procedure.^9^ ^10^ Specimens were directly embedded en bloc in TissueTek OCT (Miles Diagnostics, Elkhart, Indiana, USA) and subsequently snap-frozen in liquid nitrogen.

### Immunohistochemistry

From each frozen tissue block serial sections (5 μm) were cut and stained with the following mouse monoclonal antibodies: anti-CD3 (SK7; Becton Dickinson), anti-CD4 (SK3; Becton Dickinson) and anti-CD8 (DK25; Dako, Glostrup, Denmark) to detect T cells, anti-CD22 (CLB-B-ly; Central Laboratory of the Netherlands Red Cross Blood Transfusion Service, Amsterdam, the Netherlands) to detect B cells, anti-CD68 (EBM11; Dako) to detect macrophages, anti-CD55 (clone 67; Serotec, Oxford, UK) to detect fibroblast-like synoviocytes, anti-CD138 (clone B-B4; Immunotech, Marseille, France) to detect plasma cells and anti-CD23 (clone MHM6; Dako) as well as anti-CD21L (a kind gift from Dr Y Liu, Schering-Plough, Dardilly, France) to detect follicular dendritic cells (FDCs), as described previously.^4^ ^11^ ^12^ Sections with non-assessable tissue, defined by the absence of an intimal lining layer, were not analysed. For control sections, the primary antibodies were omitted or irrelevant antibodies were applied.

To determine the distribution of B cells and FDCs in lymphocyte aggregates, we evaluated two separate tissue sections (each section representing one level of the six biopsy samples that were embedded en bloc), located 50 μm apart, which were stained with the anti-CD22, anti-CD23 and anti-CD21L antibodies. CD22 rather than CD19 was used to detect B cells in tissue, as it results in more reliable staining on the tissue level. The presence of FDCs was assessed by CD23 staining and subsequently confirmed by CD21L staining.

### Digital image analysis

All sections were coded and randomly analysed by computer-assisted image analysis using a Leica DM-RXA light microscope and a program written in the programming language QUIPS with specialised Qwin software (both from Leica, Cambridge, UK).^13^ ^14^ An independent observer (Marjolein Vinkenoog, Division of Clinical Immunology and Rheumatology of the Academic Medical Centre/University of Amsterdam, The Netherlands) who was unaware of the clinical data performed the acquisition and image analysis. For all markers, 18 high-power fields were analysed, as previously described and validated.^15^ CD68 expression was analysed separately in the intimal lining layer and the synovial sublining. CD22 expression was analysed on two levels as described above, and the mean number of B cells was used for statistical analysis. Data are expressed as the number of cells per mm^2^, representing cell density.

Lymphocyte aggregates were counted and classified as previously described.^16^ Aggregates with a radius of 2–5 cells were defined as grade 1, those with a radius of 5–10 cells were graded as 2, and those with a radius of >10 cells were graded as 3. The total number of grade 1, 2 and 3 was added up per section. The resulting figure was termed the total number of aggregates.

### Statistical analysis

Changes in the DAS28 were compared using the Student paired t test. Changes in peripheral blood lymphocytes, synovial cell populations, as well as serum levels of total IgM, total IgG, IgM-RF and ACPA were compared using Wilcoxon signed rank test for paired data. A mixed linear model was used as a repeated measurements method to confirm the results of the separate paired t test for analysis of changes in synovial parameters.

Correlations between changes in synovial cell populations, serum IgM-RF and ACPA levels and the DAS28 between baseline, 4 weeks and 16 weeks after treatment were determined by Spearman correlation coefficient. Differences in baseline synovial cell populations and changes in cell populations between responders and non-responders to treatment were determined by the Mann–Whitney U test for unpaired data.

For analysis of possible predictive biomarkers for clinical response we calculated the Spearman correlation coefficient between the change in DAS28 (between baseline and week 24) and changes in B cells, T cells, plasma cells and intimal and sublining macrophages (between baseline, 4 weeks and 16 weeks). For dichotomous analysis response was determined as a decrease in DAS28 of ⩾1.2 at 24 weeks^17^ and also as according to the European League Against Rheumatism (EULAR) response criteria.^18^

The Mann–Whitney U test for unpaired data was used to compare the clinical response in patients with FDCs or lymphocyte aggregates at baseline compare to those without. Parameters that were significantly related to the decrease in DAS28 were subsequently analysed by univariate linear regression analysis to assess the predictive value. Finally, these parameters were analysed together with multiple linear regression analysis in a backward model.

## RESULTS

### Clinical and demographic features

Clinical and demographic details are shown in table 1.

**Table 1:** Baseline characteristics of the study patients

Demographic	
Median age, years (range)	55 (22–75)
Female, no. (%)	18 (75)
Disease status:	
Median disease duration, years (range)	12 (0.9–50)
Erosive disease, no. (%)	24 (100)
Nodular disease, no. (%)	9 (38)
Median IgM-RF, kU/litre (IQR)	75 (46–159)
Median ACPA, kU/litre (IQR)	240 (95–1031)
Median DAS28, (IQR)	6.5 (1.1)
Median ESR, mm/h (IQR)	37 (19–55)
Median CRP, mg/dl (IQR)	24 (10–76)
Medications:	
Median no. of previous DMARDs, (IQR)	4 (2–5)
Median no. of previous biological agents, (IQR)	2 (1–3)
Median methotrexate dosage, mg/week (IQR)	15 (10–25)
Corticosteroids, no. (%)	16 (67)
Median prednisone dosage, mg/day (IQR)	5 (0–10)

ACPA, anti-cyclic citrullinated peptide; CRP, C-reactive protein; DAS28, 28-joint Disease Activity Score; DMARDs, disease-modifying antirheumatic drugs; ESR, erythrocyte sedimentation rate; IQR, interquartile range; RF, rheumatoid factor.

### Clinical response to treatment with rituximab

One patient withdrew from the study after 20 weeks because of insufficient clinical response. For analysis related to response at week 24 the observations from her week 20 visit were carried forward. The DAS28 was unaltered at 4 weeks (peri-infusional corticosteroids were not allowed), but there was a significant decrease in DAS28 at 16 and 24 weeks (compared to baseline: mean (SD) decrease of 1.6 (1.1) and 1.6 (1.6), respectively; both p<0.001). At 24 weeks 16 of the 24 patients (67%) had a decrease in DAS28 of at least 1.2. In all, 3 patients (13%) had a good response according to the EULAR response criteria, 14 patients (58%) a moderate response and 7 patients (29%) did not fulfil the EULAR response criteria.

### Decreased IgM-RF and ACPA levels after rituximab treatment

The IgM-RF and ACPA levels were not available for the week 24 visit and week 36 for the patient who withdrew from the study after 20 weeks because of insufficient clinical response. For another patient the visit at week 36 was not performed because of personal circumstances. Thus, serial IgM-RF and ACPA levels were available for 22 patients.

**Figure 1:**
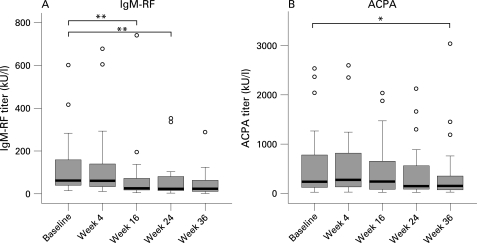
Change in levels of IgM-rheumatoid factor (IgM-RF; A) and anti-citrullinated peptide antibodies (ACPA; B) before and during the 36 weeks after initiation of rituximab treatment. The circles represent outliers (values of more than 1.5 box lengths from the upper or lower edge of the box; the box length is the interquartile range). *p<0.05, **p<0.01.

There was a highly significant decrease in serum levels of IgM-RF at 16 (p = 0.006) and 24 weeks (p<0.001) after treatment (fig 1). There was a trend towards lower ACPA levels at 24 weeks with a statistically significant decrease after 36 weeks (p = 0.015) (fig 1). At 24 weeks the serum levels of IgM-RF and ACPA decreased significantly more than those of their respective antibody classes (p = 0.001 for IgM-RF compared with total serum IgM; p = 0.026 for ACPA compared with total serum IgG levels).

### Depletion of B cells in peripheral blood

CD19+ B cells in PB were undetectable 4 and 16 weeks after rituximab treatment (table 2). Low numbers of B cells could be measured in 3/24 patients at week 4 and 4/24 patients at week 16 (0.01×10^9^/litre). At 24 weeks B cells started to return in 13 of 23 analysed patients (median 0.01×10^9^/litre (IQR 0.00–0.02). The total numbers of T cells and T cell subsets did not change significantly after treatment (table 2).

**Table 2:** Cells in peripheral blood and synovial tissue obtained from patients with rheumatoid arthritis before, 4 and 16 weeks after rituximab treatment

	Before rituximab	4 weeks after rituximab	p Value*	16 weeks after rituximab	p Value†
**Blood lymphocytes:**					
CD19	0.13 (0.09–0.19)	<0.01 (0.00–0.00)	<0.001	<0.01 (0.00–0.00)	<0.001
CD3	1.32 (0.81–1.68)	1.17 (0.90–1.90)	0.78	1.26 (1.00–1.87)	0.49
CD3CD4	0.93 (0.52–1.14)	0.78 (0.63–1.35)	0.64	0.85 (0.69–1.22)	0.66
CD3CD8	0.29 (0.19–0.48)	0.34 (0.20–0.52)	0.51	0.34 (0.22–0.49)	0.38
Cellular markers in synovial tissue:					
CD22	38 (3–158)	9 (0–39)	0.002	8 (0–40)	0.015
CD3	249 (119–845)	391 (62–1114)	0.65	130 (44–383)	0.010
CD4	403 (2–1702)	78 (15–1128)	0.92	152 (55–659)	0.068
CD8	8 (0–27)	3 (0–6)	0.67	1 (0–10)	0.048
CD138	137 (58–496)	174 (0–623)	0.71	76 (0–213)	0.48
CD68L	293 (93–502)	148 (66–346)	0.043	133 (18–215)	0.001
CD68SL	548 (134–1076)	434 (88–1291)	0.112	191 (52–563)	0.023
Aggregates	8 (0–32)	4 (0–24)	0.078	1 (0–2)	0.007

CD68+ macrophages were analysed separately in the intimal lining layer (L) and synovial sublining (SL). Values for peripheral blood lymphocytes are median (interquartile range (IQR)) 10^9^/litre, for cellular markers in synovium median (IQR) cells/mm^2^. The total no. of lymphocyte aggregates (aggregates) was counted per section.

*Wilcoxon signed rank test for paired data between baseline and week 4. †Wilcoxon signed rank test for paired data between baseline and week 16.

### The effects of rituximab are not limited to synovial B cells: changes in plasma cells, T cells, FDCs and macrophages

Out of the total of 24 patients analysed, baseline biopsies of 2 patients did not pass synovial tissue quality control; of these patients only samples taken after 4 and 16 weeks were included in the analysis. In two other patients the biopsy samples taken after 16 weeks and in one patient the biopsy taken after 4 weeks was of insufficient quality to be included in the analysis.

**Figure 2:**
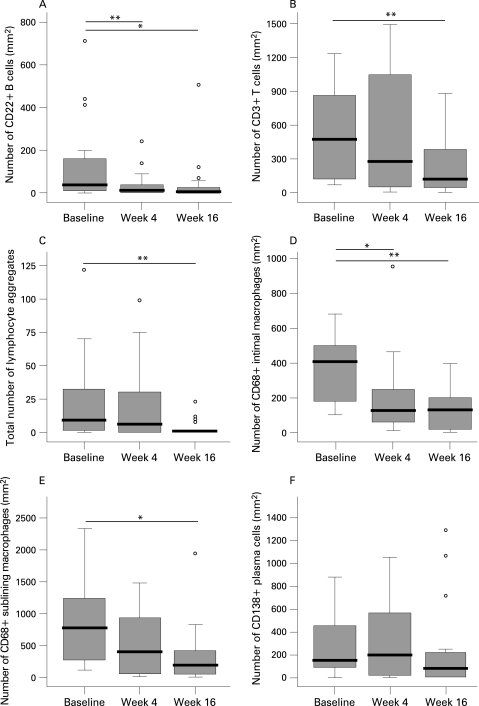
Change in number of CD22+ B cells (A), CD3+ T cells (B), lymphocyte aggregates (C), intimal (D) and sublining (E) CD68+ macrophages and CD138+ plasma cells (F) in synovial tissue. Biopsies were obtained before, 4 and 16 weeks after initiation of rituximab treatment. The circles represent outliers (values of more than 1.5 box lengths from the upper or lower edge of the box; the box length is the interquartile range). *p<0.05, **p<0.01.

Extension of the study population in the present study confirmed our previous observation,^4^ showing a highly significant reduction of synovial B cells at 4 weeks, but not in all patients (fig 2, table 2). Similar results were obtained when we used anti-CD19 antibodies to detect synovial B cells by immunohistochemistry (data not shown). There was no statistically significant additional reduction of synovial B cells at 16 weeks on the group level, but there was a clear trend towards more pronounced B cell depletion in 7 of the 15 patients who had persistent B cells at week 4 (median 39 cells/mm^2^ (IQR 12–89) and 4 (0–22), at respectively 4 and 16 weeks after rituximab infusion). In five patients there was clear persistence of B cells (median 52 (IQR 9–551) and 101 (63–313) at baseline and at 16 weeks, respectively).

Since B cells are precursors of plasma cells, and B cell depletion could indirectly result in a decrease in short-lived plasma cells associated with autoantibody production,^19^ we examined the consequences of rituximab treatment for plasma cell numbers in the synovium. There was a marked reduction of plasma cells in a subset of the patients (fig 2, table 2). On the group level this change did not reach statistical significance due to the variability of the response.

The number of T cells was unaltered at 4 weeks, but there was a significant decrease in T cell numbers at 16 weeks (fig 2, table 2). Consistent with the reduction of synovial B cells and T cells (the major cell populations in the lymphocyte aggregates), we observed a trend towards reduced numbers of lymphocyte aggregates at 4 weeks with a significant decrease of aggregates of all sizes at 16 weeks (fig 2, table 2). FDCs were found in four patients at baseline, but were undetectable in all patients 16 weeks after treatment. Hence, rituximab treatment had a clear effect on lymphocyte aggregates and germinal centres (defined by the presence of FDCs).

Of interest, we also found a significant decrease in intimal macrophages at 4 weeks, which was even more pronounced at 16 weeks (fig 2, table 2). In addition, there was a trend towards lower numbers of sublining macrophages at 4 weeks with a statistically significant reduction at 16 weeks (fig 2, table 2). Using repeated measurement methods for analysis of changes in synovial cell populations between baseline and 4 and 16 weeks after treatment yielded the same results as separate paired t test analysis.

Persistence of B cells after 16 weeks was correlated with persistence of plasma cells (r = 0.70; p<0.001), T cells (r = 0.69; p<0.001), and lymphocyte aggregates (r = 0.72; p<0.001) at the same timepoint.

### Changes in synovial plasma cells predict clinical response

Since the clinical response to rituximab treatment may be variable, we studied whether changes in synovial cell populations were related to clinical improvement.

**Figure 3:**
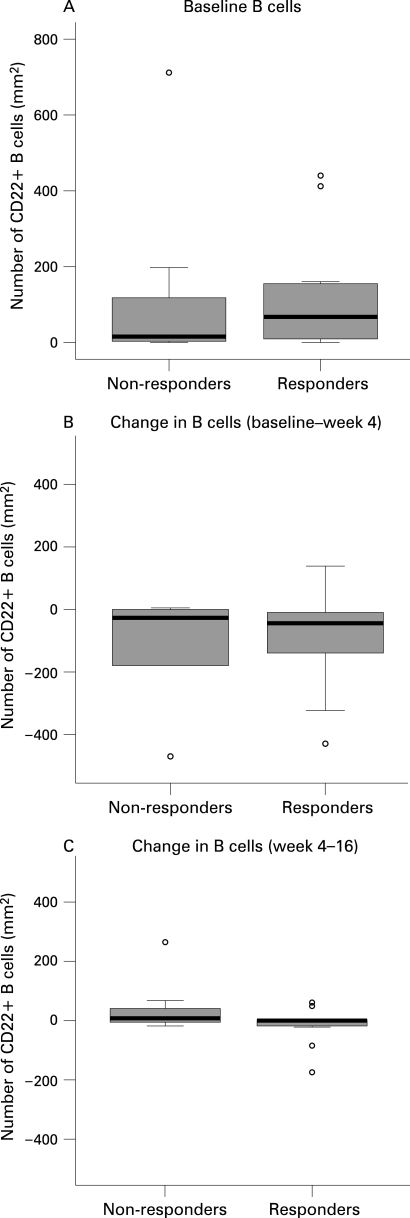
Differences in synovial B cells at baseline (A), respectively changes in B cells after rituximab treatment (B and C), in clinical responders versus non-responders. There was no statistically significant difference in B cell numbers at baseline or in the reduction in synovial B cells between responders and non-responders. The circles represent outliers (values of more than 1.5 box lengths from the upper or lower edge of the box; the box length is the interquartile range). *p<0.05, **p<0.01.

**Figure 4:**
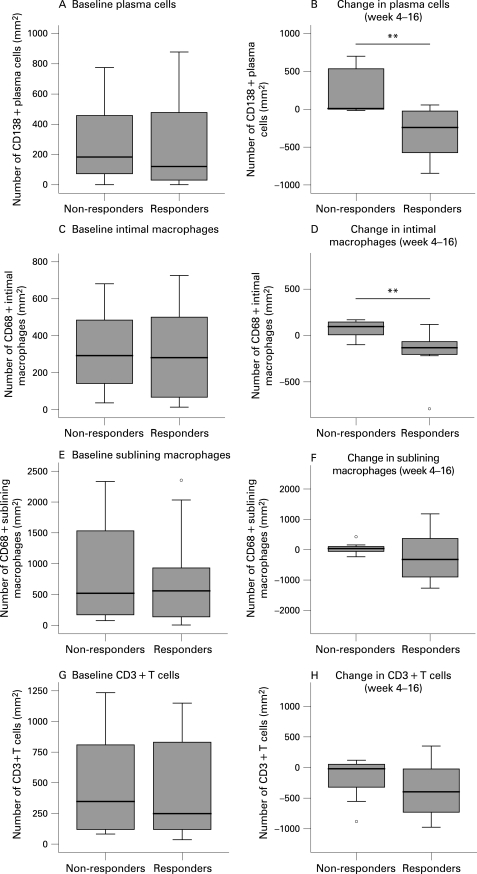
Differences in synovial plasma cells (A), intimal macrophages (C), sublining macrophages (E) and T cells (G) at baseline respectively changes in these cells (B, D, F, H) after rituximab treatment in clinical responders versus non-responders. There was no statistically significant difference in these cells at baseline between responders versus non-responders. In light of the kinetics of the changes after treatment (fig 2), we compared the decrease in synovial cell populations other than B cells between 4 weeks and 16 weeks in relationship to clinical response. There was a highly significant difference in reduction of intimal macrophages (p = 0.008), respectively plasma cells (p = 0.002), between responders compared to non-responders with a similar trend for sublining macrophages. The circles represent outliers (values of more than 1.5 box lengths from the upper or lower edge of the box; the box length is the interquartile range). *p<0.05, **p<0.01.

Of importance, there were no baseline characteristics of the synovium that could significantly predict clinical response to treatment, although there was perhaps a minor trend towards more B cells at baseline in responders compared to non-responders (figs 3 and 4). The decrease in synovial B cells between baseline and 4 weeks or between 4 weeks and 16 weeks was not significantly different between responders versus non-responders to treatment.

**Figure 5:**
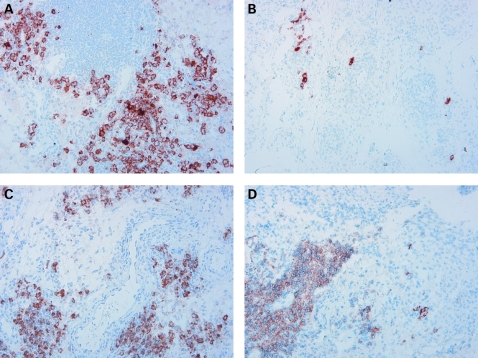
Change in the number of CD138+ plasma cells in representative serial synovial tissue samples obtained at 4 (A and C) and 16 (B and D) weeks after initiation of rituximab treatment. Different patterns of response were identified. In patients who responded to treatment we observed a reduction in plasma cells between 4 and 16 weeks after treatment (compare A and B), while in patients who did not fulfil the response criteria, plasma cells persisted (compare C and D) (Original magnification × 20). Linear regression analysis revealed a significant relationship between the decrease in plasma cell numbers and the decrease in 28-joint Disease Activity Score (DAS28) at week 24.

Additionally, no changes in other inflammatory cells between baseline and 16 weeks after treatment differed between responders and non-responders, although a trend was found for plasma cells (p = 0.115). As described above, the changes in synovial cells other than B cells were found between 4 and 16 weeks rather than between baseline and 4 weeks, except for intimal macrophages where there was already a reduction at 4 weeks (fig 2). In light of the kinetics of the changes shown in table 2 and fig 2, we compared the decrease in synovial cell populations other than B cells between 4 weeks and 16 weeks in relationship to clinical response (fig 4). Of interest, the change in plasma cells differed significantly between responders and non-responders (p = 0.002) (fig 4B and 5). Within the clinical responder group there was a significant decrease in plasma cells after treatment (p = 0.005), but not so in non-responders. Similarly, there was a significant difference in reduction of intimal macrophages between responders and non-responders (p = 0.008; fig 4D), with a similar trend for sublining macrophages (fig 4F).

Linear regression analysis was performed to establish the predictive value of the changes in synovial cell populations for the size of the clinical response after 24 weeks. Consistent with the analyses described above, we found a positive correlation between the change in intimal macrophages (r = 0.51; p = 0.04) as well as plasma cells (r = 0.46, p = 0.003) between 4 and 16 weeks on the one hand and the decrease in DAS28 after 24 weeks on the other. Accordingly, linear regression analysis revealed that the decrease in plasma cells between 4 and 16 weeks could predict the decrease in DAS28 at 24 weeks after treatment (R^2^ = 0.26, p = 0.002). The reduction of macrophages also showed a trend for predicting clinical improvement at week 24 in the univariate analysis (p = 0.051), but when analysed together with the decrease in plasma cells in a multiple linear regression model, it was not an independent predictor of the decrease in DAS28 (p = 0.216, partial correlation 0.399).

When linear regression analysis was performed on the combination of changes in plasma cells between 4 and 16 weeks and the baseline DAS28 using multiple linear regression analysis, the decrease in plasma cells continued to predict the decease in DAS28 at week 24 (p = 0.034), whereas the baseline DAS28 alone could not predict the clinical response (p = 0.623). Interestingly, the change in plasma cell numbers was also correlated with a decrease in the ACPA levels at week 16 (r = 0.52, p = 0.03).

When we used the change in synovial cells between baseline and 16 weeks, similar trends were observed. As noted above and shown in table 2 and fig 2, the major changes in synovial cell populations other than B cells were observed between 4 and 16 weeks after treatment, secondary to the changes in synovial B cells.

Collectively, the results suggest that the clinical response can be predicted by changes in cell types other than B cells, especially the number of synovial plasma cells that are derived from B cells. This change is also correlated to the reduction in serum ACPA levels.

## DISCUSSION

The results presented here confirm the previously reported variable tissue response of B cells after rituximab treatment, in contrast to the nearly complete depletion of B cells in the peripheral blood. Moreover, this study shows for the first time the secondary effects on cell populations other than synovial B cells, supporting the concept that B cells orchestrate synovial inflammation. In particular the change in short-lived synovial plasma cells (derived from B cells) between 4 and 16 weeks after initiation of treatment is related to the clinical response over time. These findings are consistent with the kinetics of the gradual clinical response and the slow but sure decrease in levels of circulating antibodies observed after rituximab treatment.

B cells may drive the inflammatory processes involved in RA by different mechanisms. First, B cells may drive synovial inflammation by production of autoantibodies;^20^ they are the precursors of short-lived plasma cells associated with production of autoantibodies, such as IgM-RF and ACPA. Second, B cells are effective antigen-presenting cells and activators of T cells.^21^ Third, B cells may promote synovial inflammation by producing pro-inflammatory cytokines and chemokines.^22^^–^24 Thus, depletion of B cells could interfere with different mechanisms involved in the pathogenesis.

The results from the present study show that rituximab treatment may indeed deplete B cells at the primary site of inflammation, the synovium, although there is persistence of synovial B cells in a subset of patients. The discrepancy with the complete B cell depletion observed in peripheral blood in nearly all patients might be explained by the expression of protective factors in the tissue, such as BLyS^25^ and CD55 (decay-accelerating factor (DAF)),^26^ as well as the requirement for B cells to access the circulation for efficient depletion. This difference underscores the importance of analysis of different compartments to understand the effects of treatment, as has also been shown after, for example, Campath-1H treatment.^27^ The variable response in the synovial tissue with regard to B cell depletion suggests that the standard therapeutic regimen is perhaps not optimal in all patients. Of note, persistence of synovial B cells was related to persistence of plasma cells. Future studies need to address the question whether it is possible to induce clinical improvement in non-responders with persistent B cells and plasma cells in the synovium. Conceivably, a subset of patients would benefit from a more intense dosing schedule. It is also possible that persistence of plasma cells in non-responders is related to the presence of long-lived plasma cells in the synovium of a subset of patients. Plasma cells with different longevity could be induced by mechanisms such as epitope spreading. For these patients alternative approaches may be considered, interfering with, for example, APRIL (a proliferation-inducing ligand) and B lymphocyte stimulator (BLyS). The present study strengthens the rationale for evaluating changes in biomarkers after targeted therapies interfering with B cells and plasma cells to further optimise the clinical response in the context of personalised medicine.

The relationship between the change in plasma cells and clinical improvement suggests that rituximab exerts its effects at least in part by an indirect effect on short-lived autoreactive plasma cells that are associated with the production of autoantibodies. Consistent with these results, there was a reduction in RF and ACPA levels after treatment; the reduction in plasma cells was directly related to the decrease in ACPA levels at week 16. These data are also in agreement with a previous study showing a trend towards lower synovial immunoglobulin synthesis 2 months after rituximab treatment, although that study was not powered to detect statistically significant changes.^5^ A role for autoantibodies in RA has been suggested since the discovery of RF in RA and regained interest in the late nineties.^20^ The previously reported decrease in RF and ACPA levels after rituximab treatment relative to minor changes in total immunoglobulins suggests a role for short-lived plasma cells in their production.^2^ ^3^ ^28^ This notion is supported by observations in a mouse model; rheumatoid factor transgenic mice were crossed with mice of the autoimmune-prone MRL/lpr strain, after which a spontaneous rheumatoid factor response developed. This response was mediated by continuous generation of short-lived plasmablasts.^19^ In addition, it has been suggested that small immune complexes containing autoantibodies may drive synovial inflammation by triggering Fcγ receptor IIIA, which is expressed by intimal macrophages.^29^ The results presented here would support the concept that autoantibody production by B cells and plasma cells is critically involved in promoting synovial inflammation.

Depletion of B cells did not only indirectly result in a decrease in synovial plasma cells, but there was also an effect on other major cell populations, such as T cells and macrophages. This indicates that B cells have an important role in sustaining the inflammatory cell infiltrate in the rheumatoid synovium. The decrease in synovial T cells and the disruption of lymphocyte aggregates and germinal centres, as shown by the disappearance of FDCs, supports the hypothesis that B cells influence T cell activity and organisation in the synovial tissue. Lymphocyte aggregates could be disrupted by the absence of B cell derived factors such as lymphotoxin beta.^30^ However, it is quite likely that the explanation is more complex, since previous work has shown that, in contrast to large follicles, relatively small lymphocyte aggregates usually contain very few B cells.^31^ Interestingly, B cell depletion also diminished macrophage infiltration, which is in agreement with the concept that there is a consistent relationship between clinical improvement and changes in synovial macrophages, independent of the primary mechanism of action of the treatment.^32^ ^33^ Together, the change in T cells and macrophages could be explained by an indirect effect of B cell depletion on the expression of proinflammatory cytokines and chemokines involved in cell migration and retention, although this remains to be shown.

In conclusion, rituximab treatment results in a variable response on synovial B cells with secondary changes in numbers of other inflammatory cells, leading to diminished synovial inflammation. There is a direct relationship between the decrease in synovial plasma cells and clinical improvement over time.
